# Single-cell RNA sequencing of aging neural progenitors reveals loss of excitatory neuron potential and a population with transcriptional immune response

**DOI:** 10.3389/fnins.2024.1400963

**Published:** 2024-08-09

**Authors:** Jonas Fritze, Stefan Lang, Mikael Sommarin, Shamit Soneji, Henrik Ahlenius

**Affiliations:** ^1^Stem Cells, Aging and Neurodegeneration Group, Faculty of Medicine, Department of Experimental Medical Science, Lund University, Lund, Sweden; ^2^Lund Stem Cell Center, Lund, Sweden; ^3^Computational Genomics Group, Faculty of Medicine, Division of Molecular Hematology, Lund University, Lund, Sweden; ^4^Stem Cells and Leukemia Group, Faculty of Medicine, Division of Molecular Hematology, Lund University, Lund, Sweden

**Keywords:** neurogenesis, aging, intermediate progenitors, neuroblasts, immune response, dentate gyrus, subventricular zone, excitatory

## Abstract

In the adult murine brain, neural stem cells (NSCs) can be found in two main niches: the dentate gyrus (DG) and the subventricular zone (SVZ). In the DG, NSCs produce intermediate progenitors (IPs) that differentiate into excitatory neurons, while progenitors in the SVZ migrate to the olfactory bulb (OB), where they mainly differentiate into inhibitory interneurons. Neurogenesis, the process of generating new neurons, persists throughout life but decreases dramatically with aging, concomitantly with increased inflammation. Although many cell types, including microglia, undergo significant transcriptional changes, few such changes have been detected in neural progenitors. Furthermore, transcriptional profiles in progenitors from different neurogenic regions have not been compared on a single-cell level, and little is known about how they are affected by aging-related inflammation. We have generated a single cell RNA sequencing dataset enriched for IPs, which revealed that most aged neural progenitors only acquire minor transcriptional changes. However, progenitors set to become excitatory neurons decrease faster than others. In addition, a population in the aged SVZ, not detected in the OB, acquired major transcriptional activation related to immune responses. This suggests that differences in age related neurogenic decline between regions is not due to tissue differences but rather cell type specific intrinsic transcriptional programs, and that subset of neuroblasts in the SVZ react strongly to age related inflammatory cues.

## 1 Introduction

In the adult murine brain, quiescent neural stem cells (NSCs) can be found in two main niches: the subgranular zone (SGZ) of the dentate gyrus (DG), which lies in the hippocampus, and the subventricular zone (SVZ) lining the lateral ventricles (Jurkowski et al., 2020). Upon activation, neural stem cells (NSCs) self-renew and/or differentiate to produce intermediate progenitors (IPs), called type-2 cells in the SGZ and transit-amplifying progenitors (TAPs) in the SVZ, which have the potential to develop into various cell types of the brain (Bonaguidi et al., 2016). To generate neurons, early IPs develop into neuroblasts, another IP state, which differentiate into immature neurons before transforming into fully mature neurons. In the DG, neuroblasts migrate to the granule cell layer where they primarily differentiate into excitatory granule neurons, while neuroblasts in the SVZ migrate via the rostral migratory stream to the olfactory bulb (OB) where they mainly mature into inhibitory interneurons (Lledo et al., 2006).

Neurogenesis, the process by which NSCs generate neurons, persists throughout life but decreases drastically with age. The NSC pools become smaller and lose functionality with aging, leading to a decline in the proliferation of IPs, which results in fewer newly formed neurons (Katsimpardi and Lledo, 2018). Consequently, neurogenesis-dependent functions, such as olfactory discrimination and pattern separation, decline with aging (Alonso et al., 2006; Breton-Provencher et al., 2009; Lazarini et al., 2009; Moreno et al., 2009; Johnston et al., 2016; Dillon et al., 2017). Simultaneously, brain inflammation increases with microglia, which are the immune cells of the brain, reacting to aging (Wendimu and Hooks, 2022) and peripheral immune cells infiltrating the central nervous system (Dulken et al., 2019).

Single-cell sequencing has been used to study different cell types during aging. Many cell types, including microglia, undergo dramatic transcriptional changes; however, few such changes have been detected in IPs, likely because their relatively low numbers (Ximerakis et al., 2019) make their analysis challenging. While NSCs have been studied extensively in the context of aging, IPs from different regions have not been compared, and little is known about how inflammation and aging affect the transcriptional integrity of neuroblasts and the potential for new neurons to integrate in the aged brain.

We generated a single-cell RNA sequencing dataset enriched for IPs from the SVZ, OB, and DG of adult, middle-aged, and aged mice. The dataset revealed that most IPs acquire only minor transcriptional changes. Additionally, it showed that neuroblasts set to become excitatory neurons decrease faster than other neuroblast populations, and a neuroblast population in the aged SVZ, not found in the OB, acquires major transcriptional activation related to immune responses.

## 2 Materials and methods

### 2.1 Animals

All experimental procedures were approved by the Malmö–Lund Ethical Committee for the use of laboratory animals and were conducted following the European Union directive on animal rights. Mice expressing a green fluorescent protein (GFP) under the Dcx promoter (Dcx-GFP) (Couillard-Despres et al., 2006), which is highly active in neuroblasts, were bred and housed in the animal facility connected to the Lund University Biomedical Center under sterile conditions in individually ventilated cages (IVCs) at 22°C, with 40%−60% humidity and a 12-h light/dark cycle with *ab libitum* access to food and water. Adult (3–4 months), middle-aged (12–16 months), and aged (18–24 months) mice were used, with male and female mice equally distributed between groups. Tissues from four to five mice were pooled for each age group.

### 2.2 Brain dissection and tissue dissociation

Heterozygote Dcx-GFP mice were euthanized by cervical dislocation, and their brains were kept in an ice-cold L-15 medium (Invitrogen: Waltham, Massachusetts, United States), as previously described (Ahlenius and Kokaia, 2010). To facilitate the comparison of neurogenic niches, the OBs were isolated and the SVZs and DGs were dissected from 1-mm sections separately. Tissue from 4-5 mice were pooled in ice-cold L-15 medium separately for each region (from the same group of animals) and dissociated into a single -cell suspension using the Adult Brain Dissociation Kit, mouse and rat (Miltenyi Biotech: Bergisch Gladbach, North Rhine-Westphalia, Germany, 130-107-677) following the manufacturer's guidelines. The cells were then purified through a two-step gradient as previously described (Ahlenius and Kokaia, 2010) and resuspended in fluorescence-activated cell sorting (FACS) medium [4% Bovine serum albumin (BSA) (W/V), 2% 2M 4-(2-hydroxyethyl)-1-piperazineethanesulfonic acid (HEPES) (V/V), and 0.1% Sodium azide (V/V) in Hanks' balanced salt solution (HBSS) (BSA, HEPES and HBSS: Thermo fisher; Waltham, Massachusetts, United States)].

### 2.3 Fluorescence-activated cell sorting

To enrich for neural progenitors, dissociated cells from Dcx-GFP mice were subjected to fluorescence-activated cell sorting (FACS; Supplementary Figure 1a) for separation based on their endogenous GFP expression, and cell viability was assessed with propidium iodide using FACSARIAII/III (BD Biosciences: Franklin Lakes, New Jersey, United States). The sorted cells were collected into Phosphate-buffered saline (PBS) with 2% Fetal bovine serum (FBS) for single-cell sequencing library preparation on the same day. Alternatively, these cells were collected into QIAzol lysis reagent (Qiagen: Venlo, Netherlands (corporate) Hilden, Germany (operational)) and stored at −80°C for bulk sequencing.

### 2.4 RNA extraction

For bulk RNA sequencing, total RNA was isolated from the sorted GFP+ cells using the miRNeasy Micro Kit (Qiagen) according to the manufacturer's instructions. Briefly, the samples frozen in the QIAzol lysis reagent were brought to room temperature and homogenized by pipetting, and the RNA was then separated with a chloroform phase gradient. During RNA purification, on-column DNase digestion (Qiagen) was performed, and the final RNA was eluted into 14 μl of H_2_O. Concentrations were measured using a bioanalyzer (Agilent: Santa Clara, California, United States) and loaded using the RNA 6000 Pico Kit (Agilent).

### 2.5 Library preparation and sequencing

For single-cell RNA sequencing, libraries were generated from FACS-sorted cells expressing endogenous GFP (Dcx-GFP) for each experimental group separately using a Chromium system (10 × Genomics: Pleasanton, California, United States), according to the manufacturer's guidelines [Single Cell 3′ Reagent Kits v2 User Guide (CG00052)], with 12–13 cDNA amplification cycles and 14 sample index cycles. The libraries generated from 2,100 aged DG cells, 3,000 aged OB cells, and 5,000 cells for all other samples were sequenced on a NextSeq 500 System (Illumina: San Diego, California, United States) using NextSeq 500/550 High Output v2 Kits (Illumina). Each sample contained cells from four to five mice. The cells from different regions but of the same age group were obtained from the same group of animals.

Bulk RNA sequencing libraries were prepared using the SMARTer Stranded Total RNA-Seq Kit v2, Pico Input Mammalian (Takara Bio USA, Inc.) according to the manufacturer's user manual (063017). Furthermore, 2 ng of total RNA was used from each sample with 4-min fragmentation time and 14 PCR cycles for amplification of the final library in a Mastercycler X50s (Eppendorf: Hamburg, Germany). The libraries were sequenced on a NextSeq 500 System (Illumina) using NextSeq 500/550 High Output v2.5 Kits (Illumina). The sequencing was performed at the Center for Translational Genomics, Lund University, and Clinical Genomics Lund, SciLifeLab. Each biological sample (*n* = 5) contained cells from four to five mice for the bulk data analysis. The cells from all different regions were collected from every animal included in the study.

### 2.6 Raw data processing and quality control

Raw data from single-cell RNA sequencing were processed through the Cell Ranger pipeline to generate count matrices, which were then loaded into the R computational environment (4.1.2) using read10xCounts (DropletUtils) and merged into a single-cell experiment object (i.e., SingleCellExperiment). The counts were normalized using logNormCounts (Scater) with size factors based on quickCluster and computeSumFactors (Scran). Then, the logcounts were extracted as dgCMatrices and merged into a Seurat object using CreateSeuratObject (Seurat). The low-quality cells with fewer than 75 or more than 4,000 detected genes (features), more than 1.5% mitochondrial genes, or more than 15% ribosomal genes were discarded (Supplementary Figure 1b). A very low number of cells expressing high levels of Cx3cr1 and Iba1, which are markers for microglia, were also discarded.

For bulk sequencing, FASTQ files were organized using bcl2fastq2 with default settings and without warnings from FastQC (Supplementary Figures 2a–c). Reads were aligned to the mouse reference genome (GRCm38) from the Ensemble database using HISAT2 (Supplementary Figures 2d, e). Quality control of the aligned data, including base distribution and insert size checks, was performed using Picard (Supplementary Figure 2a). Assembly of the alignments into full transcripts and quantification of expression levels were performed using StringTie.

### 2.7 Clustering and cell type assignment

**Figure 1:**
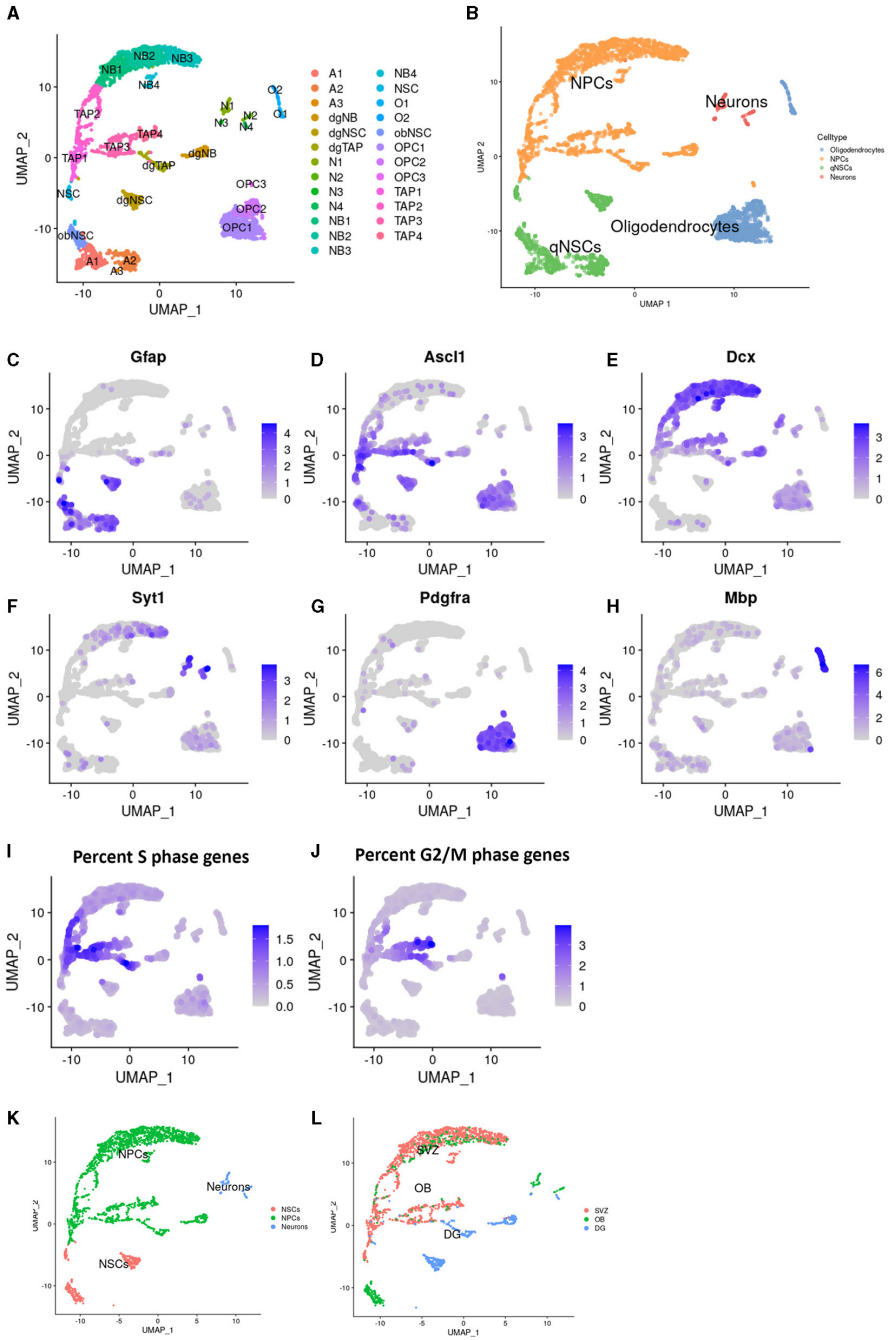
Identification of populations in Dcx+ cells sorted from neurogenic niches. Uniform manifold approximation and projection (UMAP) plots showing cluster names **(A)** and cell type annotation **(B)**. Visualization of common markers, in the UMAP, for Astrocytes and NSCs; Gfap **(C)**, TAPs; ASCl1 **(D)**, Neuroblasts; Dcx **(E)**, Neurons; Syt1 **(F)**, OPCs; Pdgfra **(G)**, and Oligodendrocytes; Mbp **(H)**. Percentage of genes prominently expressed in the s-phase **(I)** and g2- and m- phase of the cell cycle **(J)**. Cell type annotation after cell filtering **(K)** and regional identity **(L)** in the UMAP.

The data analysis was performed in the R computational environment (4.1.2). All genes were scaled with ScaleData (Seurat) to accurately compare the expression between cells. The 2,000 most highly variable genes (HVGs; Supplementary Figure 1c) were identified using the variance stabilizing transformation (VST) method via FindVariableFeatures (Seurat), which were then used for the principal component analysis. The first 20 principle components with the highest standard deviation were selected manually from an elbow plot and used for Louvain clustering (Seurat) with a k parameter of 10 and a resolution of 1 (Figure 1A). Clusters were visualized with uniform manifold approximation and projection (UMAP), initially annotated using singleR (1.8.1) based on a reference mouse brain dataset from Celldex (Benayoun et al., 2019) (Figure 1B), and further adjusted manually to commonly used markers (i.e., Glial fibrillary acidic protein (Gfap) for astroglial and Neural stem cells (NSCs), Achaete-scute family bHLH transcription factor 1 (Ascl1) for NSCs and early progenitors, Doublecortin (Dcx) for neuroblasts, Synaptotagmin 1 (Syt1) for neurons, Platelet derived growth factor receptor alpha (Pdgfra) for oligodendrocyte precursors, and Mbp for mature oligodendrocytes; Figures 1C–H). The clusters assigned as oligodendrocytes were discarded, along with the clusters where more than 40% of the population expressed Gfap and < 40% expressed Ascl1, which were considered astroglial populations without activated NSCs. DimPlot (Seurat) was used to visualize the clusters, while FeaturePlot (Seurat) was used to illustrate gene expression.

### 2.8 Differential gene expression analysis and quantifications

For single-cell RNA sequencing data, differential gene expression analysis was performed using findMarkers (Seurat), considering *p*-values based on Bonferroni correction, using all genes in the dataset; the adjusted *p*-values (*p*_val_adj) of < 0.05 are considered statistically significant. ComplexUpset was used for upset plots, Ggplot (ggplot2) for boxplots and extreme point plots, and DoHeatmap (Seruat) for heatmaps. Gene ontology (GO) terms were identified for differentially expressed genes (DEGs), with the adjusted *p*-values refers to how the DEGs were chosen, while other settings refers to the enrichGO function from ClusterProfiler (4.2.2), with parameters set to minGSSize 1, maxGSSize 500, and both qvalue Cutoff and pvalueCutoff were set to 1. GraphPad Prism v7 was used to visualize population size estimation data.

For bulk RNA sequencing data, DESeq2 was used for differential gene expression (DGE) analysis, which compares adult samples to a combined group of middle-aged and aged samples. Heatmaps were generated using pheatmap, color pallets were created using RColorBrewer, and bar graphs were created using GraphPad Prism v7. Inflammatory response genes can considerably vary due to infection or other external stimuli; therefore, to identify outliers, two independent statistical tests were conducted: Cooks' distance (Supplementary Figures 3a, b) and Grubbs' test (Supplementary Table 1). One adult sample was identified as an outlier for inflammation-related genes in both tests and was not included in the DGE analysis for inflammatory response genes. The DGE analysis included four or five biological replicates, each pooled with the RNA of four to five adult mice. Furthermore, 10 middle-aged and aged mice were included in the DGE analysis.

Cell numbers were normalized to OPCs since it is the population in our data set most stable during aging and present in large enough quantity.

## 3 Results

### 3.1 Aged neuroblasts display minor but cell state dynamic transcriptional changes

NSCs are fewer in number and less active in the aged brain compared to the adult mammalian brain. This decline leads to the production of fewer IPs and, ultimately, fewer new neurons added to the brain during aging (Katsimpardi and Lledo, 2018). Although recent research has revealed age-related changes in stem cells, little is known about how IPs such as neuroblasts are affected by aging.

To study the effect of aging on IPs, including TAPs and neuroblasts, in the two major neurogenic niches, we dissected the SVZ, OB, and DG from 3-, 14-, and 24-month-old Dcx-GFP mice. The GFP+ cells were isolated using FACS and single-cell RNA sequencing was performed using 10 × and Illumina chemistry. During data processing, we performed unbiased clustering of cells from all ages and regions, which were digitally pooled together (Figure 1A). Clusters were annotated computationally based on previous RNA sequencing studies (Figure 1B) and manually verified and further specified using common markers. Cells in clusters with high expression of both Gfap and Ascl1 were annotated as NCSs, with Ascl1 alone as TAPs, Dcx as neuroblasts, Syt1 as mature neurons, Pdgfra as oligodendrocyte progenitors, and Mbp as mature oligodendrocytes (Figures 1C–H). The expression of cell cycle genes (Figures 1I, J) highlighted highly proliferative cells, such as TAPs.

We identified all major common cell types involved in neurogenesis with a strong enrichment of IPs. Interestingly, some clusters split into substates that were not previously described (Figure 1A), indicating that enrichment, by FACS, for neural progenitors aided in revealing their full heterogeneity.

Clusters with cells from neuronal lineages were selected for further downstream analysis (Figure 1K). We identified two differentiation trajectories from NSCs to mature IPs in the SVZ (Supplementary Figure 1d), one leading to IPs with a potential to generate inhibitory neurons and one to excitatory neurons as previously described (Sequerra et al., 2013). We identified a unique NSC population in the OB, as previously reported (Defterali, 2021), initiating a trajectory leading to inhibitory neurons (Supplementary Figure 1e). In the DG, we identified a single trajectory leading to excitatory neurons (Supplementary Figure 1f).

As expected, from previous histological studies (Jurkowski et al., 2020), the cells from the DG distinctly separated from most cells in the SVZ or OB, and the majority of mature cells could be detected only in the OB or DG. Most cell types at various maturity states identified in the SVZ were also detected in the OB, albeit to a lesser extent, and only a few SVZ and OB cells were clustered with DG cells (Figure 1L).

**Figure 2:**
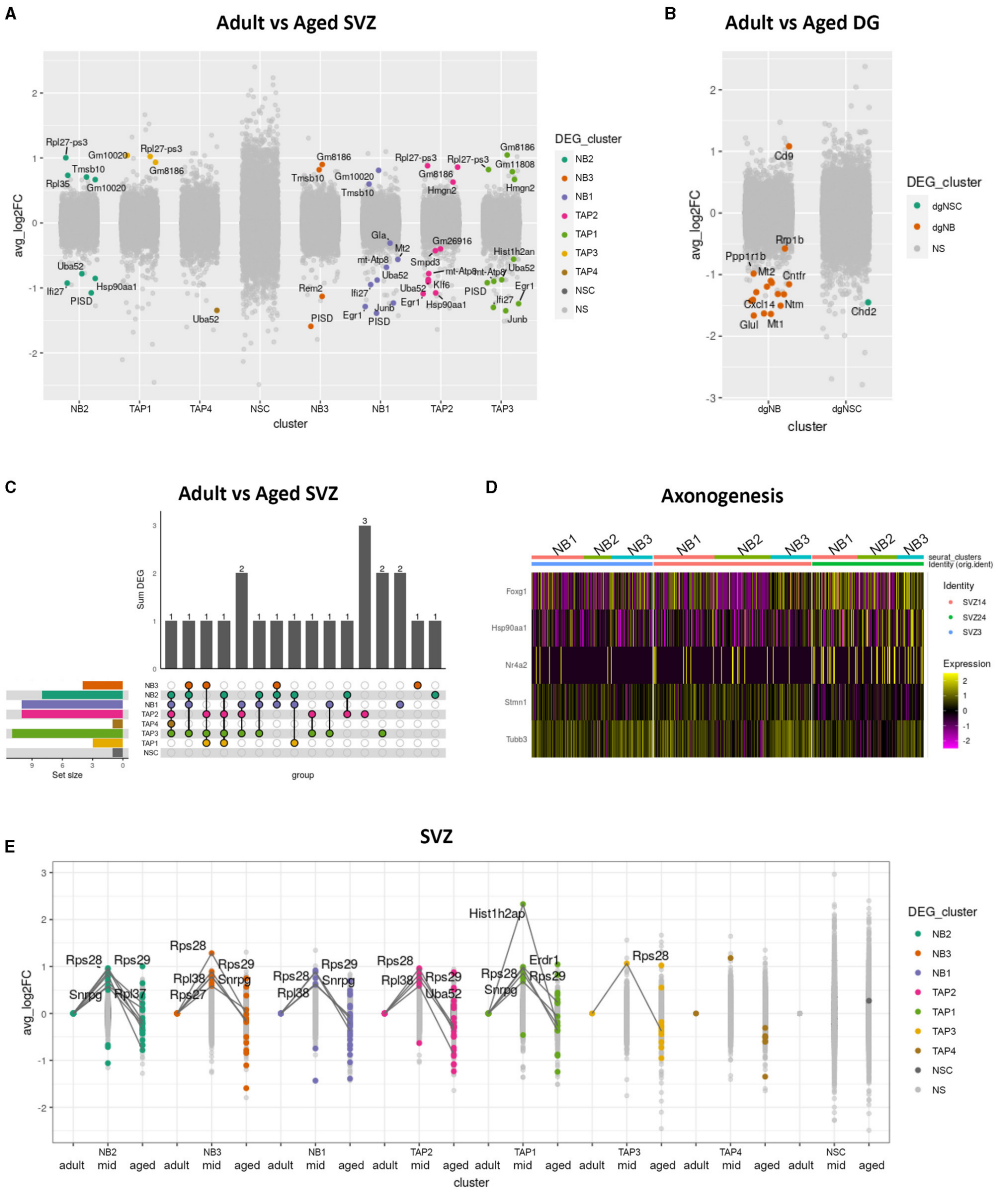
Differential gene expression in aged neurogenic niches. Boxplot showing differentially expressed genes (*p*_val_adj < 0.05) in color and average log fold changes of all genes between adult and aged clusters of the SVZ **(A)** and the DG **(B)**. Upset plot showing shared and unique differentially expressed genes between adult and aged clusters in the SVZ **(C)**. Heatmap showing differentially expressed genes between adult and aged SVZ clusters NB1, NB2, and NB3, combined for genes with the common gene ontology term “axonogenesis” **(D)**. Extreme point plot where lines highlight genes with expression max or min in the middle-aged group of different clusters in the SVZ and where colored dots indicate differential expression compared to adult **(E)**.

For each cluster where the population size remained consistent with age and there were sufficient cells in each age group to run the analysis, we performed the DEG analysis between adult and aged cells in the SVZ, OB, and DG separately (Figures 2A, B). We found only a few genes that were upregulated or downregulated in most aged cell types in the DG and SVZ and none in the OB. In the SVZ, none of these DEGs were universal for all cell types; while some were shared, others were unique. The panel of DEGs also differed between neighboring transient cell states, although a larger proportion was shared (Figure 2C).

All non-cycling neuroblast clusters (NB1, NB2, and NB3) exhibited very similar gene expression profiles (Figure 2C) and shared DEGs with age. We, therefore, decided to digitally pool them together to achieve a more powerful DEG analysis, revealing several genes involved in axonogenesis, such as Tubb3, Stmn1, and Nr4a2, to be differentially expressed between age groups (Figure 2D). Tubb3 is a major component of axons (Radwitz et al., 2022) and, along with Nr4a2 (Nurr1) and Stmn1, has been shown to promote neurite growth (Heng et al., 2012; Latremoliere et al., 2018; Kwon et al., 2020).

Next, we performed differential gene expression analysis between adult and middle-aged and between middle-aged and aged SVZ clusters. We detected several genes to have an expression maximum in the mid aged group that either dropped to, or below adult levels in the aged group (Figure 2E). This indicates that transcriptional changes occurring during aging are not always monotonic but dynamic. Many of these extreme-point genes coded for ribosomal proteins in the neuroblast clusters, while *Hist1h2ap*, a gene encoding a replication-dependent histone, was prominent in the TAP3 cluster of cycling IPs (Supplementary Figure 1b; Figure 2E).

In summary, aging leads to only a few differentially expressed genes in IPs, with some unique to specific cell types and some shared, but none universal. Despite the small changes, our data indicate that transcriptional changes during aging can be non-monotonic and that aged neuroblasts differentially express genes involved in axon development.

### 3.2 Excitatory neuron progenitors in the SVZ and DG decrease faster during aging than inhibitory neuron progenitors

NSCs in the adult DG primarily form excitatory neurons, while SVZ neurogenesis mainly produces inhibitory interneurons (Lledo et al., 2006). Having established that DG and SVZ IPs have different gene expression profiles and rarely share aging-related transcriptional changes, we next assessed if aging affects neural progenitor heterogeneity.

**Figure 3:**
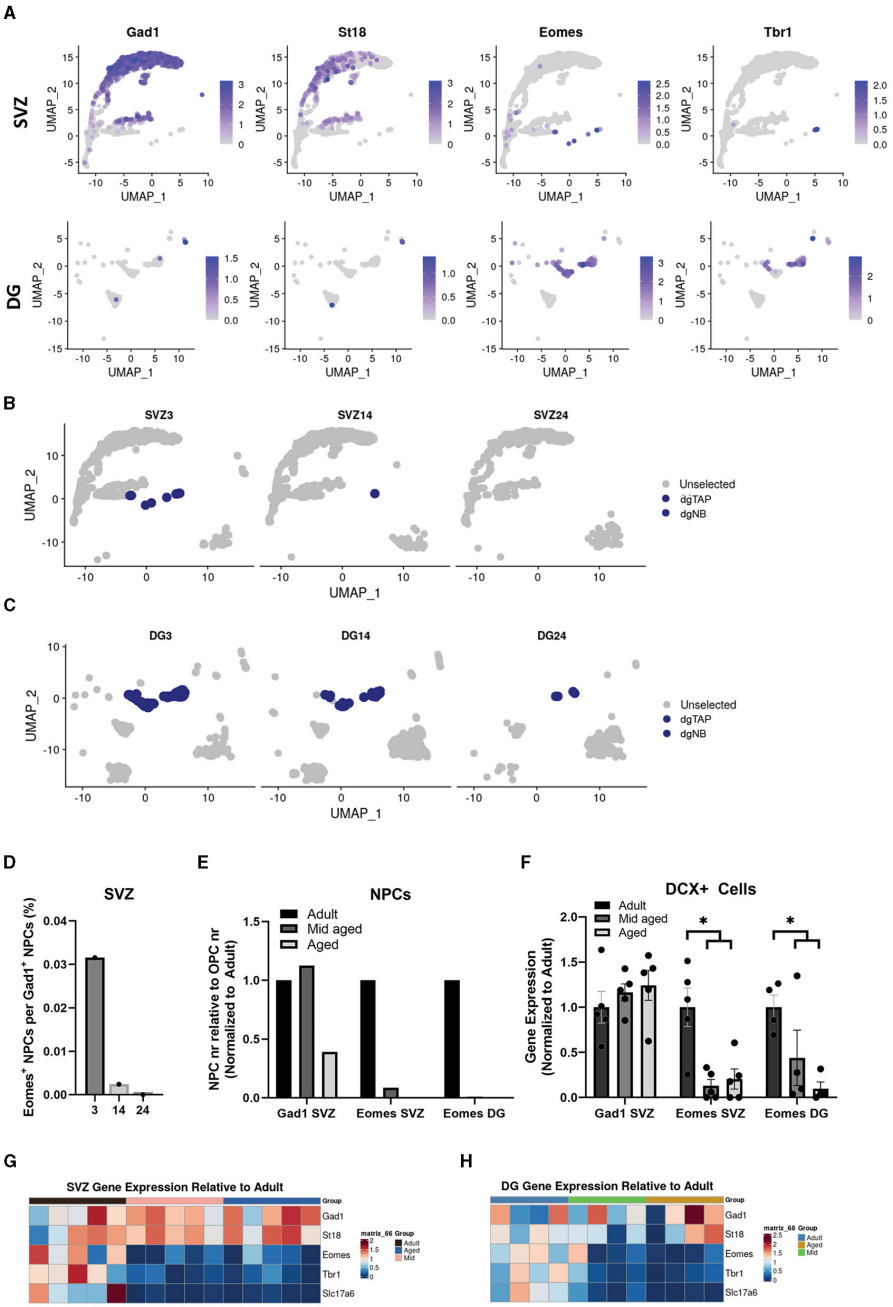
Excitatory neuron progenitors in the SVZ and DG decrease faster during aging than inhibitory neuron progenitors. Uniform manifold approximation and projection (UMAP) showing expression of common markers for inhibitory neurons, Gad1 and St18, and for excitatory neurons, Eomes and Tbr1, in the SVZ and DG **(A)**. Cluster dgTAP and dgNB highlighted in different age groups of the SVZ **(B)** and DG **(C)**. Quantification of cells in non-cycling cell populations expressing either Eomes (cluster dgTAP and dgNB) or Gad1 (cluster NB1, NB2, and NB3) in the SVZ, represented as a ratio **(D)**, and in the SVZ and DG represented as relative to OPCs and normalized to adult **(E)**. Gene expression normalized to adult of Gad1 and Eomes in bulk sequencing samples, *Adjusted *p*-value of < 0.05, with DESeq2, of adult samples compared to a combined group of middle-aged and aged samples **(F)**. Heatmap showing gene expression of common markers for inhibitory neurons, Gad1 and St18, and for excitatory neurons, Eomes, Tbr1, and Slc17a6, from different bulk samples relative to mean adult in the SVZ **(G)** and DG **(H)**.

To investigate IP heterogeneity during aging, we used clusters generated from digitally pooled samples (Figure 1A) and identified neural progenitors fated for different neuronal subtypes based on previously identified genes. We used Gad1 (Gad67) and St18 [identified here, and known to regulate inhibitory neuron fate (Nunnelly et al., 2022)] to highlight IPs set to become inhibitory interneurons and Eomes (Tbr2), Tbr1 and Slc17a6 (Vglut2) for IPs set to become excitatory neurons. Excitatory markers were clearly expressed in DG IPs in contrast to the majority of SVZ IPs that instead expressed Gad1. However, when separating the two neurogenic regions, we found that a small fraction of SVZ IPs clustered with DG IPs and expressed markers for excitatory neurons (Figure 3A). This previously described subpopulation of neural progenitors (Sequerra et al., 2013) has not been well studied in the context of aging.

The number of OPCs has shown to remain fairly constant during aging (Wang et al., 2020), while other large populations in our dataset varied. We, therefore, used OPCs as a reference to analyze how the SVZ and DG IP populations changed in size with aging. Interestingly our single cell data suggested that the population of Eomes (Tbr2) positive IPs, in both neurogenic regions, decreased more rapidly with age compared to IPs positive for Gad1 (Figures 3B–E).

To test this statistically, we sorted GFP-positive cells from adult, middle-aged, and aged SVZ of Dcx-GFP mice, performed bulk mRNA sequencing, and analyzed markers specific for the different IP populations. Our bulk data, indeed, showed that the expression of Eomes (Tbr2), Tbr1, and Slc17a6 (Vglut2) decreased more rapidly with age in the SVZ and DG compared to Gad1 and St18 in the SVZ (Figures 3F–H).

In summary, our RNA expression data suggest that the number of IPs set to become excitatory neurons, regardless of the neurogenic region, decreases faster during aging than that of IPs set to become inhibitory interneurons.

### 3.3 A distinct population of neuroblasts in the aged SVZ acquires an expression profile related to immune responses

Having shown that most aged neural progenitors retain their expression profiles with minor differences, while the composition of neuronal progenitor subtypes changes, we next investigated whether any IPs acquire new cellular identities during aging. Concomitant with decreased neurogenesis, inflammation in the brain increases (Ferrucci and Fabbri, 2018). However, it is unknown whether aged IPs change their gene expression related to immune responses and whether such changes are global or selective.

**Figure 4:**
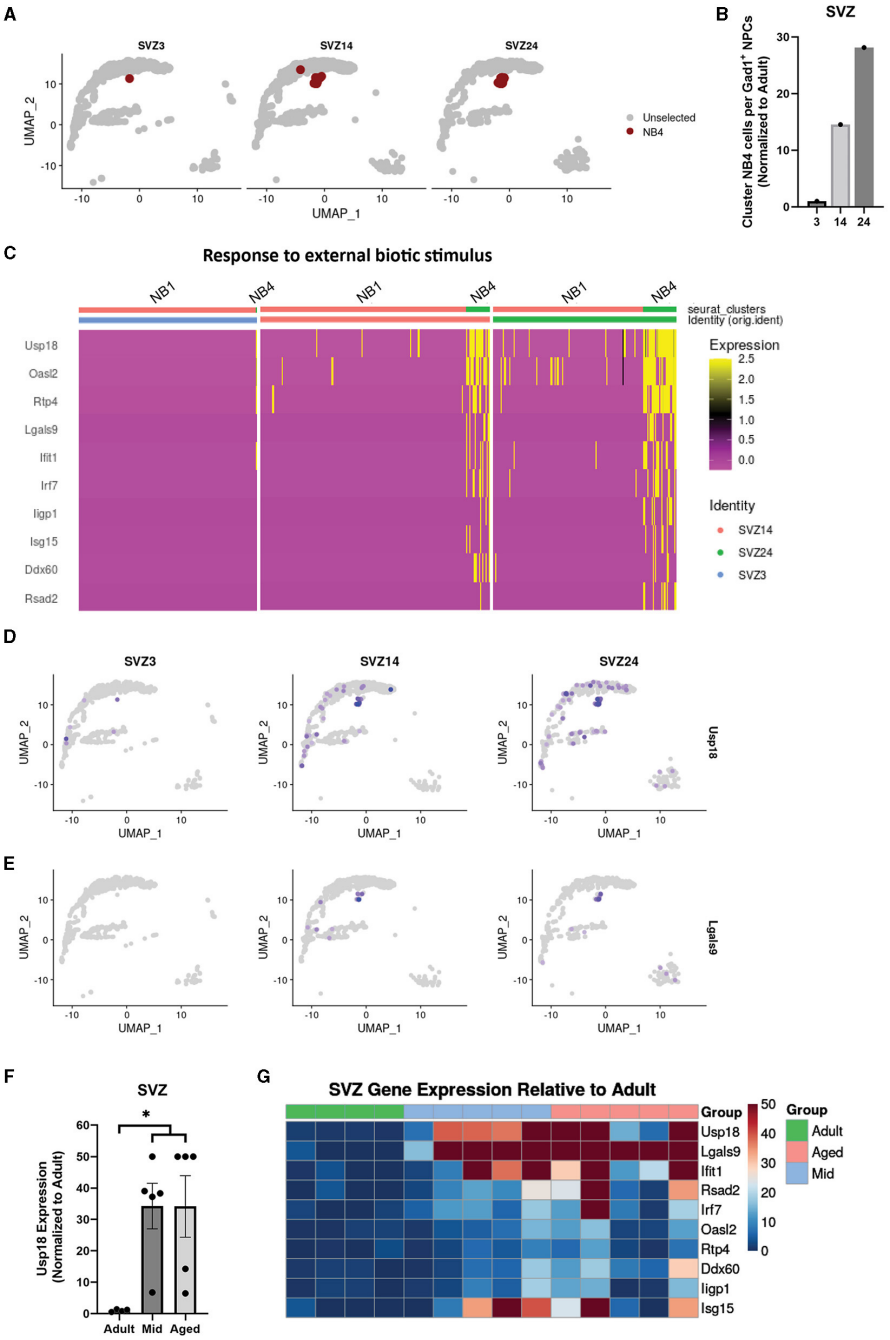
A distinct population of neuroblasts in the aged SVZ acquires an expression profile related to immune responses. Uniform manifold approximation and projection (UMAP) highlighting cluster NB4 in different age groups of the SVZ **(A)**. Quantification of cells in cluster NB4 and non-cycling cell populations expressing Gad1 (clusters NB1, NB2, and NB3) in the SVZ, represented as a ratio **(B)**. Heatmap showing differentially expressed genes between cluster NB4 and a combined cluster of NB1, NB2, and NB3 for genes with the common gene ontology term “response to external biotic stimuli,” all related to immune response **(C)**. Uniform manifold approximation and projection (UMAP) showing expression of genes defining cluster NB4 in the SVZ, Usp18 **(D)** and Lgals9 **(E)**. Gene expression of Usp18 normalized to adult in bulk sequencing samples of the SVZ, *p.adj of < 0.05, with DESeq2, of adult samples compared to a combined group of middle-aged and aged samples **(F)**. Heatmap showing gene expression of top defining genes of cluster NB4 from different bulk samples relative to mean adult in the SVZ **(G)**.

Since we did not find any major age-related gene expression differences in clusters where population size was unchanged (Figures 2A, B), we decided to analyze cluster NB4 that clearly increased in size in the SVZ with age (Figures 4A, B) and was not detectable at any age in either OB or DG. Interestingly, compared to Gad1+ IPs (NB1, NB2, and NB3), NB4 exhibited a gene expression pattern similar to neuroblasts but with several upregulated genes involved in innate immune responses and responses to external biotic stimuli (Figure 4C). By exploring the differentially expressed genes (Supplementary Table 1), we found Usp18 (Ubiquitin Specific Peptidase 18) to be highly enriched in cluster NB4 and also detected its presence to a greater extent in other cells from aged samples (Figure 4D). Usp18 is induced by viral infection and type I interferons and inhibits apoptosis (Malakhova et al., 2006; Diao et al., 2020). In addition, we found Lgals9 (Galectin-9), a suppressor of T-cells (Yang et al., 2021), to be specific to cluster NB4 (Figure 4E). Therefore, we analyzed Usp18 and Lgals9, along with other top genes defining cluster NB4 (Supplementary Table 1), in our SVZ bulk data and found an increased expression of most of these immune response genes in the SVZ from the middle-aged and aged samples compared to those in adult samples (Figures 4F, G).

In summary, a large portion of IPs in the aged neurogenic niches exhibit only minor changes in gene expression that can be linked to immune responses, while a small selection of IPs in the SVZ not detected in the OB acquire a distinct expression profile related to immune responses.

## 4 Discussion

In this study, we generated a unique dataset and performed the first detailed transcriptional analysis of highly enriched IPs from the primary murine neurogenic niches during aging. The single-cell sequencing data are supported by bulk sequencing data to include biological variance; however, they did not yield individual animal resolution due to the challenges in obtaining a sufficient number of purified IPs from single animals.

We demonstrated that most aged IPs undergo only minor transcriptional changes, some of which are non-linear, cell-type specific, or shared, but none are universal. In addition, we found that neuroblasts set to become excitatory neurons decreased faster than IPs set to become inhibitory neurons in both neurogenic niches. Furthermore, some progenitors in the aged SVZ, not present in the OB or adult animals, showed a strong immune reactive expression profile.

Aging is a heterogeneous process that varies between individuals (Somel et al., 2006; Lowsky et al., 2014) and has been reported to drive transcriptional paths differently between cell types (Ximerakis et al., 2019). We found that aging affect gene expression differently not only between cell types but also between transient cell states of neuroblasts, albeit to a lesser extent. This indicates that the currently active transcriptional program is responsible for determining which genes are vulnerable to aging-induced expression changes. Epigenetic regulation could potentially explain these findings as DNA methylation, histone modification, chromatin remodeling, non-coding RNA regulation, and RNA modification all participate in the aging process (Wang et al., 2022) and have the potential to project transcriptional changes differently, based on the currently active program.

As anticipated based on the previous literature, we observed differentiation trajectories from NSCs to excitatory neurons in the DG, to both inhibitory and excitatory neurons in the SVZ, and only to inhibitory neurons in the OB. The NSC population and the subsequent differentiation to inhibitory neurons in the OB represent an intriguing area of study because it has received less attention. This process might be differentially affected by aging as compared to neurogenesis occurring in the SVZ. However, our experiments were designed to analyze neuroblasts, not NSCs and neurons; therefore, our data do not have enough power to make any solid conclusions regarding OB NSCs, which should be addressed in separate studies.

Interestingly, we found a few genes, mainly ribosomal, that exhibited non-monotonic changes during aging, with maximum expression in the middle-aged group of several cell types in the SVZ. The increased expression of ribosomal genes in the middle-aged group reverted to adult levels in aged animals. Alterations in the ribosomes in middle age can potentially lead to a translation imbalance, contributing to the development of aging phenotypes. Such patterns can also be an attempt to rescue or compensate for aging phenotypes before they become detrimental.

A previous study of the SVZ used bulk RNA sequencing to detect non-monotonic changes during aging. This study found that the expression of most genes exhibited such patterns, with both the minimum and maximum levels of gene expression occurring at 18 months (Apostolopoulou et al., 2017). However, bulk sequencing reflects cell type abundance and expression of the same gene in different cell types, which can be regulated in different directions during aging (Ximerakis et al., 2019). Our data confirm that non-monotonic changes can occur but suggest that they are less common and not often universal.

In most aged SVZ neuroblasts, we detected only a few transcriptional changes, some of which were involved in axonogenesis. This finding suggests that most aged neuroblasts still have the potential to generate functional neurons, possibly with altered axon development. Supporting this observation, others have reported that new DG neurons can integrate into the old brain, albeit with compromised dendrite development (Trinchero et al., 2017). Among the upregulated genes in aged neuroblasts, Foxg1 suppresses differentiation (Martynoga et al., 2005) and decreases with maturation (Miyoshi and Fishell, 2012), while Tubb3, which is downregulated in aged neuroblasts, promotes differentiation (Cao et al., 2017) and increases with maturation until it peaks (Hausrat et al., 2021). This observation suggests slower maturation of aged SVZ neuroblasts compared to their adult counterparts. Foxg1 has also been reported to assist in directing toward certain neuron subtypes through Nr4a2 (Ba et al., 2023) and to shift toward a GABAergic inhibitory neuron identity (Mariani et al., 2015). This suggests that neuroblasts in the aged SVZ may produce different composition of neuron subtypes compared to adult neuroblasts.

NSCs in the adult DG and SVZ predominantly produce different types of neurons (Jurkowski et al., 2020). However, it has been suggested that a minor population of spatially defined NSCs in the SVZ give rise to excitatory neurons (Sequerra, 2014) similar to those generated in the DG. Our data showed that IPs of excitatory neurons decrease more drastically in both neurogenic regions, as previously indicated in the DG (Galvan and Jin, 2007). This finding suggests that the differences in aging-related neurogenic decline between the SVZ and that DG are not due to major regional tissue differences but are rather due to cell-type specific and dependent on cell-intrinsic transcriptional programs. Future studies should assess whether SVZ NCSs that give rise to excitatory progeny are more similar to DG NSCs, whether their activity decreases or changes as they age, whether they start generating progenitors with inhibitory potential, or whether neuroblasts with excitatory potential are pushed toward an inhibitory identity in the aged SVZ. As our observations are based purely on single-cell transcriptomic changes, which were validated using bulk data, it is important to develop robust methods to detect these and other discrete populations at the protein level *in vivo*.

While most IPs only acquired minor changes with age, we found a population of neuroblasts that displayed a strong immune-responsive gene expression profile appearing in the middle-aged and aged SVZ. This population is defined by Lgals9 expression, which is increased in all middle-aged and aged biological SVZ samples. This population was not detected in the OB, which strongly suggests that these neuroblasts, for unknown reasons, are inhibited from reaching their destination and mature into new neurons. This process could potentially be a mechanism to select for healthy neuroblasts, yet it could also be an adverse reaction due to an altered inflammatory milieu in the neurogenic niche.

While most IPs only underwent minor changes with age, we found a population of neuroblasts in the aged SVZ displaying a strong immune-responsive gene expression profile that appeared in middle-aged and aged samples. This population, defined by Lgals9 expression, was increased in all middle-aged and aged SVZ samples and was not detected in the OB, suggesting that these neuroblasts are inhibited from reaching their destination and maturing into new neurons. This inhibition could potentially be a mechanism to select for healthy neuroblasts but could also be an adverse reaction to an altered inflammatory milieu in the neurogenic niche.

It has been previously reported that an inflammatory environment modulates NSC stemness through TNF-α (Belenguer et al., 2021). TNF-α expression increases in the aged neurogenic niche, contributing to reduced neurogenesis (Fonseca et al., 2016). We did not observe any signs of altered TNF-α signaling in neuroblasts; instead, our data indicated altered interferon signaling, which, like TNF-α, is suggested to preserve stemness and restrict differentiation (Sato et al., 2020; Ibañez et al., 2023). Although limited data exist on neuroblasts, the observed interferon-related immune response may have a similar effect, contributing to fewer neuroblasts differentiating into mature neurons. In addition, we cannot exclude the possibility that the observed effects on neuroblasts are mediated through TNF-α signaling on parental NSCs, which is carried over to differentiating neuroblasts.

The immune response in neuroblasts could be triggered by increased infiltration of immune cells from the periphery. Supporting this idea, T-cell infiltration into the SVZ increases with age (Dulken et al., 2019; Zhang et al., 2022b), and Lgals9 acts as a suppressor of T-cells (Yang et al., 2021). This likely contributes to the reduced number of new neurons in the aged OB. It would be interesting to experimentally ablate infiltrating immune cells, specifically T-cells, to analyze if the reactive neuroblast population still appears in the aged SVZ. Furthermore, understanding whether this population is long-lived and has functional implications on neighboring cells is crucial. Previous studies have shown that removing senescent cells can lead to rejuvenation (Zhang et al., 2022a), making it appealing to further study and attempt to deplete this immune-reactive neuroblast population.

## Data availability statement

The data presented in the study are deposited in the Gene Expression Omnibus (GEO) repository, accession number GSE261459, which can be found at: https://www.ncbi.nlm.nih.gov/geo/query/acc.cgi?acc=GSE261459.

## Ethics statement

The animal study was approved by Malmö/Lund Ethics Committee on Animal Testing at the Lund District Court. The study was conducted in accordance with the local legislation and institutional requirements.

## Author contributions

JF: Writing – original draft, Writing – review & editing, Conceptualization, Data curation, Formal analysis, Funding acquisition, Investigation, Project administration, Resources, Validation, Visualization. SL: Conceptualization, Data curation, Formal analysis, Writing – original draft, Writing – review & editing. MS: Investigation, Writing – original draft, Writing – review & editing. SS: Data curation, Resources, Writing – original draft, Writing – review & editing. HA: Conceptualization, Funding acquisition, Project administration, Resources, Supervision, Writing – original draft, Writing – review & editing.

## Supplementary material

Supplementary Figure 1 Experimental design for single-cell RNA sequencing, showing Dcx+ IPs in the DG, SVZ, and OB neurogenic regions of adult, middle-aged, and aged mice and sorting of Dcx+ cells before library preparation and sequencing **(a)**. Violine plots showing cells retained after quality control, with (from left) more than 75 and < 4,000 detected genes, < 3,500 molecules, < 1.5% mitochondrial genes, and < 15% ribosomal genes **(b)**. Highly variable genes used for clustering **(c)**. Trajectories, from immature to mature states, of cells from the neuronal linage in the SVZ **(d)**, OB **(e)**, and DG **(f)**.

Supplementary Figure 2 Barplot showing total reads per strand per sample **(a)**, percentage of read duplication **(b)**, percentage of GC content **(c)**, and the total number of paired reads, number of reads aligned one time, and more than one time **(d)**.

Supplementary Figure 3 Boxplot showing Cook's test for marker genes for excitatory neurons **(a)**, and genes connected to inflammation **(b)**, with larger numbers indicating outliers.

Supplementary Table 1 Grubbs' test for genes connected to inflammatory responses and markers for excitatory neurons. The sample (row title) shows the sample farthest from the mean and considered an outlier when *p*-value is < 0.05.
