# Supplementary figures and images for: Single-cell RNA sequencing of aging neural progenitors reveals loss of excitatory neuron potential and a population with transcriptional immune response
Source: Front Neurosci. 2024 Aug 9;18:1400963. doi: 10.3389/fnins.2024.1400963 (PMC11341460; doi:10.3389/fnins.2024.1400963)

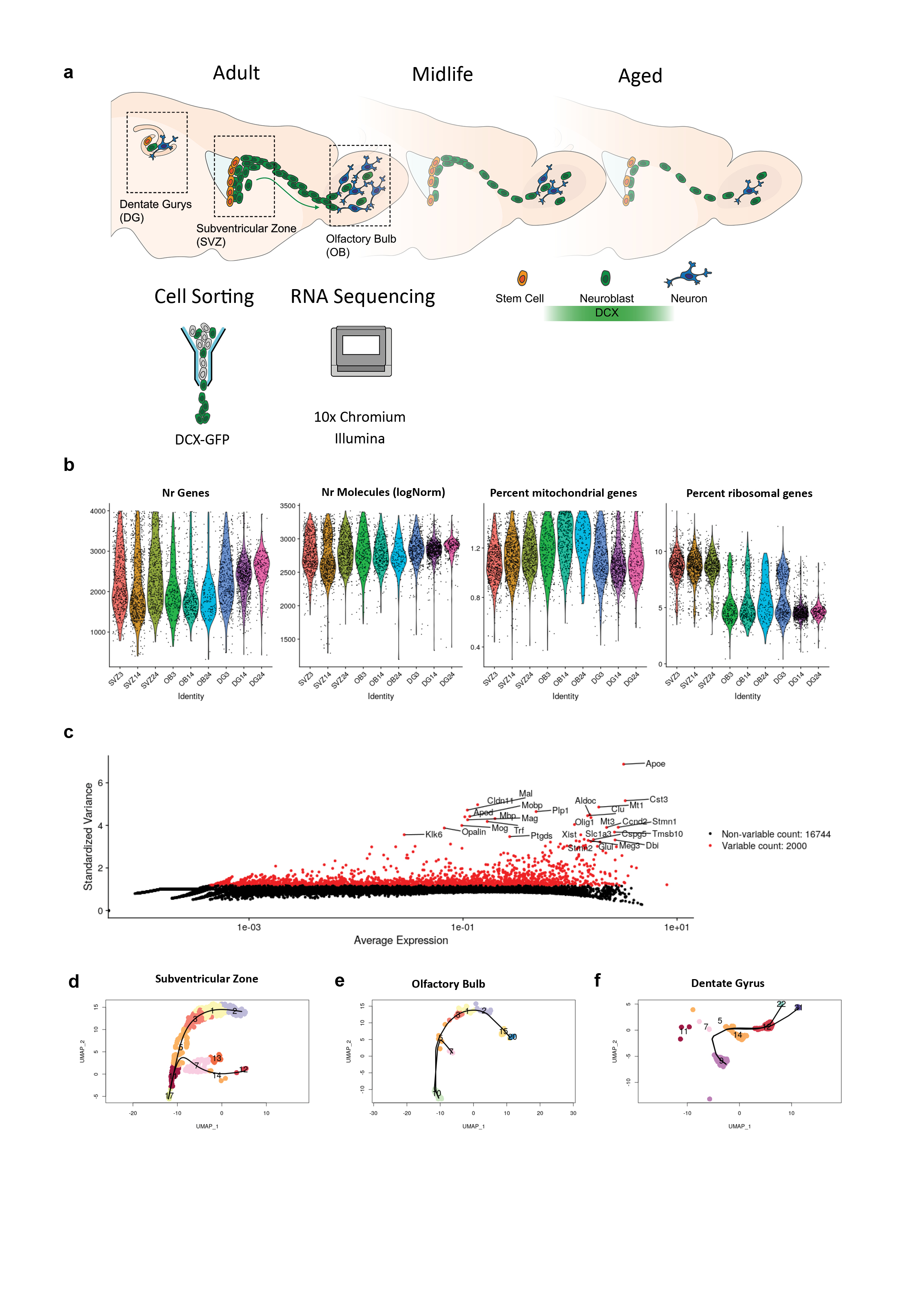

Supplement: Supplementary Figure 1 — Experimental design for single-cell RNA sequencing, showing Dcx+ IPs in the DG, SVZ, and OB neurogenic regions of adult, middle-aged, and aged mice and sorting of Dcx+ cells before library preparation and sequencing (a). Violine plots showing cells retained after quality control, with (from left) more than 75 and < 4,000 detected genes, < 3,500 molecules, < 1.5% mitochondrial genes, and < 15% ribosomal genes (b). Highly variable genes used for clustering (c). Trajectories, from immature to mature states, of cells from the neuronal linage in the SVZ (d), OB (e), and DG (f). [file Image_1.JPEG]

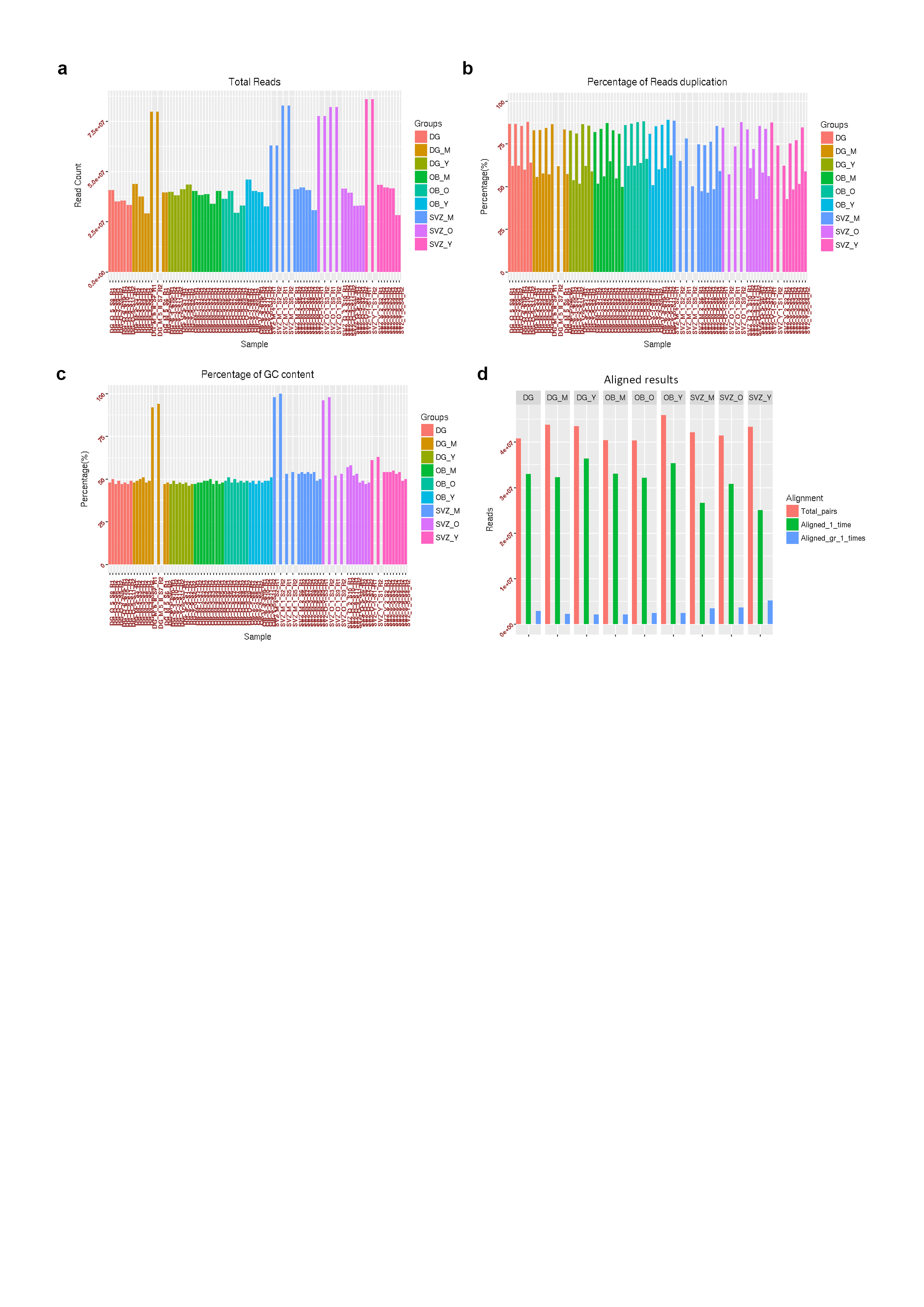

Supplement: Supplementary Figure 2 — Barplot showing total reads per strand per sample (a), percentage of read duplication (b), percentage of GC content (c), and the total number of paired reads, number of reads aligned one time, and more than one time (d). [file Image_2.JPEG]

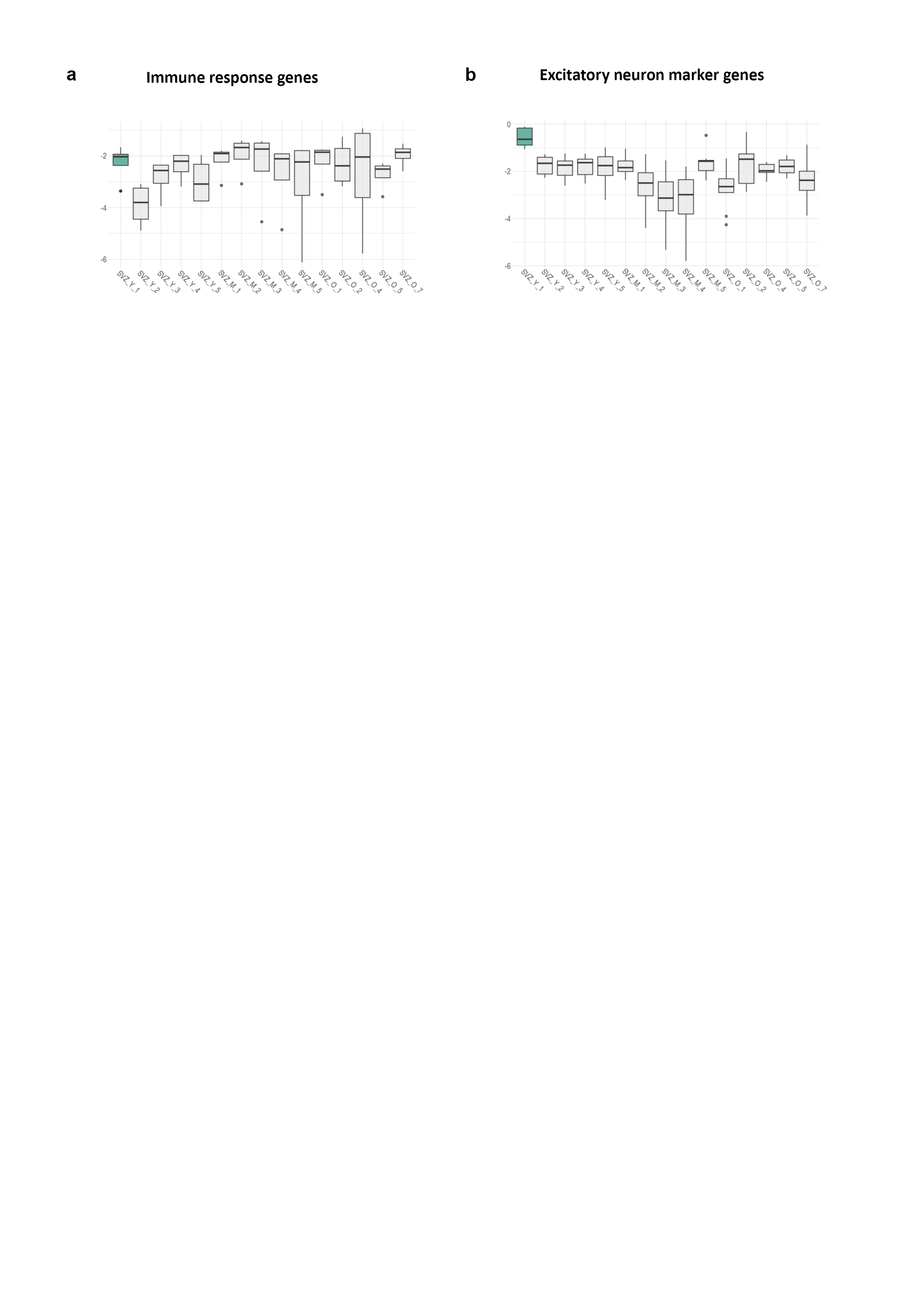

Supplement: Supplementary Figure 3 — Boxplot showing Cook's test for marker genes for excitatory neurons (a), and genes connected to inflammation (b), with larger numbers indicating outliers. [file Image_3.JPEG]
